# Identification of Soil Bacterial Isolates Suppressing Different *Phytophthora* spp. and Promoting Plant Growth

**DOI:** 10.3389/fpls.2018.01502

**Published:** 2018-10-18

**Authors:** Sharifah Farhana Syed-Ab-Rahman, Lilia C. Carvalhais, Elvis Chua, Yawen Xiao, Taylor J. Wass, Peer M. Schenk

**Affiliations:** ^1^Plant-Microbe Interactions Laboratory, School of Agriculture and Food Sciences, The University of Queensland, Brisbane, QLD, Australia; ^2^Queensland Alliance for Agriculture and Food Innovation, The University of Queensland, Brisbane, QLD, Australia

**Keywords:** biocontrol agent, microbial biofertilizer, microbial biopesticide, oomycete, *Phytophthora*, plant growth-promoting bacteria, soil microbial antagonism

## Abstract

Bacterial isolates obtained from the rhizosphere of *Arabidopsis* and a plantless compost potting mix was screened for anti-oomycete activity against *Phytophthora capsici*, *Phytophthora citricola*, *Phytophthora palmivora*, and *Phytophthora cinnamomi*. Three out of 48 isolates exhibited more than 65% inhibition against all tested *Phytophthora* species and were selected for further studies. These strains, named UQ154, UQ156, and UQ202, are closely related to *Bacillus amyloliquefaciens*, *Bacillus velezensis*, and *Acinetobacter* sp., respectively, based on 16S rDNA sequence analysis. The isolates were evaluated for their ability to fix nitrogen, solubilize phosphate, as well as for siderophore, indoleacetic acid, cell wall degrading enzymes and biofilm production. Their plant growth promoting activities were evaluated by measuring their effect on the germination percentage, root and shoot length, and seedling vigor of lettuce plants. All of these traits were significantly enhanced in plants grown from seeds inoculated with the isolates compared with control plants. Moreover, bacteria-inoculated *P. capsici*-infected chili plants exhibited improved productivity based on CO_2_ assimilation rates. Both real-time quantitative PCR and disease severity index revealed significant decreases in pathogen load in infected chili root tissues when plants were previously inoculated with the isolates. Biocontrol activity may result from the secretion of diketopiperazines as identified by Gas chromatography-mass spectrometry analysis of bacterial cultures’ extracts. Collectively, this work demonstrates the potential of bacterial isolates to control *Phytophthora* infection and promote plant growth. They can, therefore be considered as candidate microbial biofertilizers and biopesticides.

## Introduction

Food production and agricultural yields must increase worldwide to meet the demands of an increasing human population (Bommarco et al., 2013; Ibarrola-Rivas et al., 2017; Tilman et al., 2017). There is an urge for low-input and sustainable agriculture as an alternative to synthetic chemicals for controlling pests and diseases, which are the main factors driving heavy losses in agricultural yield and production (Desai and Pujari, 2014; Gupta et al., 2015; van Lenteren et al., 2018). Due to the adverse effects that chemical pesticides have on human health, the environment and other living organisms, biological control is often considered a sustainable alternative for the management of pests and plant pathogens (Chandler et al., 2011; Bach et al., 2016). There is a growing body of evidence that demonstrates the potential of manipulating leaf and root-associated microbiomes to control plant diseases and increase yields in cropping systems (Kanchiswamy et al., 2015; Santhanam et al., 2015; Bach et al., 2016; Lareen et al., 2016). To use microbes for this purpose, it is of paramount importance to understand the role of these microbes in promoting growth, especially the mechanisms of disease control, and their application as potential biopesticides.

The genus *Phytophthora* consists of 60 species, all of which are destructive plant pathogens that cause billions of dollars in losses annually in both temperate and tropical regions (Drenth and Guest, 2004; Roy, 2015). *Phytophthora cinnamomi*, a soil-borne oomycete holds a special interest due to its broad host range, mostly of woody species (Bergot et al., 2004; Cahill et al., 2008). It is considered one of the most profoundly destructive forest pathogens globally, and is responsible for severe crop diseases (Zentmyer, 1980; Shearer and Tippett, 1989). Its global spread has led to devastating consequences. For example, in Australian soils, *P. cinnamomi* is now widely distributed and causes significant devastation in not only in horticultural crops such as macadamia, pineapple and avocado but also in many endemic plant species (Cahill et al., 2008). *Phytophthora capsici* is another soil-borne oomycete that can survive in the soil for several years as oospores. This is a result of the repeated cultivation of susceptible hosts such as tomato, cucurbits, eggplant, and pepper which caused a high density of oospores in soil (Hwang and Kim, 1995). *P. capsici* has a wide range of hosts, and outbreaks of this pathogen can seriously threaten the production of cucurbits (Babadoost, 2005). Foot rot or “sudden wilt” caused by *P. capsici* is the most significant and destructive disease of black pepper which has caused losses of almost 100% in Sarawak, Malaysia (Holliday and Mowat, 1963) and 40–50% in other areas (Erwin and Ribeiro, 1996). *Phytophthora palmivora* is the global and highly damaging causal agent of several different hundreds of host plants, including ornamental, horticultural and agricultural crops (Hansen et al., 2012). The primary concern of this pathogen in a forest setting appears to be cocoa. It attacks all parts of the cocoa tree at all growth stages, often leading to 20–30% yield losses through black pod rot as well as tree deaths with up to 10% annually through stem cankers (Guest, 2007). It has been rated as the most economically important and widespread disease of cocoa with total losses due to this pathogen exceeding $400 million globally (Brasier and Griffin, 1979; Alvarez, 2015) and depending on the environmental conditions the annual losses of pod production can reach up to 90% (Bowers et al., 2001). *Phytophthora citricola* is one of the significant threats to the avocado industry and is associated with trunk, collar. and crown canker of avocado. After *P. cinnamomi*, and this pathogen has been recorded in almost 90% of the avocado orchards in California (Coffey, 1987; Marais, 2004). Furthermore, *P. citricola* has been reported as the second most destructive causal agent of root rot (Schubert et al., 1999). Each of the pathogens mentioned above has severe effects on crop production. Additional research is required to develop new effective strategies and alternatives for the management of the *Phytophthora* spp. Because it is predominately soil-borne, this pathogen is particularly hard to control. Beneficial biocontrol microbes may be a potential alternative to tackle this issue. The aim of the present study was to screen for microbial biocontrol agents with broad range of activities against *Phytophthora* spp, including *P. capsici*, infecting chili pepper. An *Arabidopsis* rhizosphere soil microbe collection was used because we hypothesized that it could be advantageous to isolate microbes from a different, but related, host where pathogens have not yet overcome resistance mediated by beneficial microbes. Firstly, we evaluated the inhibitory effects of soil bacterial isolates against *P*. *capsici*, *P. citricola, P. palmivora*, and *P. cinnamomi* in dual culture plate assays and assessed the plant growth promoting effects of these isolates *in vitro* and *in vivo*. Secondly, we investigated the effects of these isolates on CO_2_ assimilation, stomatal conductance and transpiration rate of chili plants in the presence of *Phytophthora*. Thirdly, we examined the protective effects of the isolates on chili pepper (*Capsicum annuum* L., cultivar Cayenne) plants against *P. capsici*. Our results provide evidence of the ability of these beneficial isolates to reduce *P. capsici* infection in chili. Finally, we performed a chemical characterization on bacterial cultures to discover potential anti-oomycete compounds using both mass spectrum and retention index information in GC-MS. We identified three beneficial bacterial isolates and characterized their plant growth promotion attributes and biocontrol properties against *P. capsici*, *P. citricola, P. palmivora*, and *P. cinnamomi*. We also identified six diketopiperazines (DKPs); from extracts of each of the three bacterial isolates namely hexane, dichloromethane, and ethyl acetate.

## Materials and Methods

### Screening of Bacterial Isolates for Antagonistic Activity

A subset of 48 isolates was randomly chosen from the Plant–Microbe Interactions Laboratory microbial collection at the University of Queensland for *in vitro* tests including antagonistic activity against different *Phytophthora* spp. Isolates from this collection were originally obtained from bulk, and rhizosphere soil of *Arabidopsis thaliana* Col-0 plants grown in a potting mix (Carvalhais et al., 2013).

The bacterial isolates were then tested for their ability to produce anti-oomycete metabolites against *Phytophthora* spp. according to Kumar et al. (2012) with some modifications. The screenings included the species *P. capsici*, *P*. *citricola, P. palmivora*, and *P. cinnamomi*. This test consisted of a dual-culture *in vitro* assay on potato dextrose agar (PDA, Oxoid). Standardized bacterial suspensions (OD_600nm_ of 0.1) were prepared from overnight cultures of bacterial isolates. A volume of 50 μL of each culture was inoculated symmetrically three cm away from an agar plug with mycelium placed in the center of the PDA plate. PDA plates inoculated only with an agar plug with mycelium of each *Phytophthora* species in the center served as controls. Measurements were taken after incubation at 28°C for 7 days. Percentages of inhibition were calculated using the formula (C-P/C × 100), where C is the mycelial growth (in mm) of the pathogen in the control plate (medium only), and P is the mycelial growth of the pathogen in the dual-culture. Morphologies of hyphae in the vicinity of bacterial colonies were observed under a light microscope (Model CX22; Olympus, NY, United States). Each experiment included three replicates and was repeated thrice.

### Bacterial Genomic DNA Extraction and 16S rDNA Gene Sequencing

A volume of 1.5 mL of overnight-grown cultures of each isolate were harvested by centrifugation at 11,000 *g* for 5 min and removal of the supernatant by gentle pipetting. Genomic DNA of the bacterial isolates was extracted using the ISOLATE II Plant DNA Kit (Bioline, Inc., Taunton, MA, United States) according to the manufacturer’s instructions. The isolated DNA was subjected to PCR amplification with a universal 16S primer set consisting of 27F (5′-AGA GTT TGA TCM TGG CTC AG-3′) and 1492R (5′-TAC GGY TAC CTT GTT ACG ACT-3′), targeting the almost full-length 16S rDNA gene. PCR was performed in a 25 μL reaction mixture containing: 14.75 μL ultra-pure water, 5 μL of 5X Phire buffer (Thermo Fisher Scientific), 1.25 μL of dNTPs (10 μM), 1.25 μL of 10 μM 27F primer, 1.25 μL of 10 μM 1492R primer, 0.5 μL of Phire^®^ hot-start II (Thermo Fisher Scientific), and 1 μL of DNA template (10 ng μL^−1^). The PCR Thermocycler conditions were 30 s at 98°C for initial denaturation, 30 cycles of 15 s at 98°C, 30 s at 56°C for annealing and 45 s at 72°C, followed by 7 min at 72°C for final extension. Amplifications were performed in duplicate for each sample, and the presence of the predicted PCR products was confirmed by 1% agarose gel electrophoresis. The PCR products were purified using the Wizard^®^ SV PCR Clean-Up System (Promega), and purity was confirmed using a NanoDrop One spectrophotometer. A volume corresponding to 50 ng of PCR product was added to a 12-μL reaction mixture, containing 10 pM of 27F primer and water, and then sends to the Australian Genome Research Facility Ltd., Melbourne, for sequencing by an AB3730 DNA Analyser (Applied Biosystems).

### Plant Growth Promoting Traits

#### Nitrogen Fixation

The nitrogen-fixing ability of three bacterial isolates was confirmed by growing the isolates on semi-solid nitrogen-free bromothymol blue (NFB) medium (Goswami et al., 2015). A total of 50 μL of overnight bacterial culture in YEP broth was pipetted onto the Petri plate with NFB medium and incubated in the dark at 28°C for 4 days. The nitrogen-fixing ability was determined by the color change of the medium from green to blue. Each isolate was tested with three replicates. A total of 50 μL YEP broth pipetted on the NFB medium was used as negative control. The composition of the NFB medium was (L^−1^): malate (5.0 g), KOH (4.0 g), K_2_HPO_4_ (0.5 g), FeSO_4_⋅7H_2_O (50 mg), MnSO_4_⋅7H_2_O (10 mg), MgSO_4_⋅7H_2_O (10 mg), NaCl (20 mg), CaCl_2_ (10 mg), Na_2_MoO_4_ (2 mg), 0.5% bromothymol blue alcoholic solution (2.0 mL), agar (1.75 g), distilled water (made up to 1000 mL), pH adjusted to 6.8 (Dobereiner et al., 1976). 0.5% bromothymol blue alcoholic solution was prepared by dissolving 0.5 g of bromothymol blue powder in 50 mL of 95% ethanol, then topping up with 50 mL of distilled water.

#### Phosphate Solubilization

The ability of the bacterial isolates to solubilize calcium phosphate was assayed using Pikovkaya’s medium plates containing 10 g dextrose, 0.5 g yeast extract, 5 g Ca_3_(PO_4_)_2_, 0.1 g MgSO_4_, 0.1 mg MnSO_4_, 0.1 mg FeSO_4_, 0.5 g (NH_4_)_2_SO_4_ and 15 g agar in 1 L distilled water (Pikovskaya, 1948). A loopful of each bacterial culture (OD_660nm_ of 0.1) was spot-inoculated in the center of the plate. After incubation for 48 h at 28°C, a clear zone around the colony was used as an indication for inorganic phosphate solubilization. The diameter of the halo zone and the bacterial colony was measured after 72 h. The solubilization zone was calculated by subtracting the diameter of the bacterial colony from the diameter of the total zone (Pikovskaya, 1948). Pi solubilizing index (PSI) was calculated as the diameter of halo (mm) + diameter of a colony (mm)/diameter of a colony (mm) as described by Meena et al. (2015). All tests were performed in triplicate.

#### Production of Siderophores

Production of siderophores by the bacterial isolates was assayed on medium (Sigma, Ltd.), following the method described by Schwyn and Neilands (1987). Catechol-type siderophores were measured in culture supernatants according to Arnow (1937), while hydroxamate siderophores were measured according to Csáky (1948). In the analyses, 2,3-dihydroxybenzoic acid and hydroxylamine hydrochloride, respectively, were used as standards. The bacterial suspension was prepared from a single colony obtained from an overnight culture Chromeazurol S agar streaked on a plate of Chromeazurol S agar. Ten microliters of each bacterial suspension (OD_660nm_ of 0.1) was spot-inoculated in the center of the plate and incubated at 28°C for 48–72 h. Development of a yellow-orange halo around the growth was considered positive for siderophores production. The halo diameter was measured and recorded. Each assay was performed in triplicate.

#### Indoleacetic Acid Production (IAA)

Indoleacetic acid (IAA) production was confirmed by the colorimetric method introduced by Gordon and Weber (1951) with some modifications. A total of 0.5 mL of overnight bacterial culture grown on YEP broth was centrifuged at 4,500 rpm for 5 min. The supernatant was removed, and the bacterial pellet was then inoculated in 1 mL of Luria-Bertani (LB) broth in a 1.5 mL tube supplemented with L-tryptophan at the concentration of 800 μg.mL^−1^. Components of LB broth were (L^−1^): tryptone (10.0 g), yeast (5.0 g), NaCl (5.0 g), distilled water (1 L). L-tryptophan was filter-sterilized and transferred into autoclaved LB broth using 0.22-μm syringe membrane filter. After an incubation period of 96 h in the dark at 28°C, the tube was centrifuged at 10,000 rpm for 10 min. About 0.5 mL of the supernatant was transferred into a new tube and mixed with 0.5 mL of Salkowski’s reagent (2 mL of 0.5 M FeCl_3_ solution, 49 mL of distilled water and 49 mL of 70% perchloric acid). The sample was incubated at room temperature in the dark for 30 min before absorbance at 530 nm was measured. The concentration of IAA produced by the sample was determined by comparison with an IAA standard curve ranged from 1 to 20 μg.mL^−1^. Each isolate was tested in triplicate.

#### Biofilm Production

Biofilm production by bacterial isolates was determined according to the colorimetric assay described by Erriu using 96-well microtiter plate (Erriu et al., 2012). Bacterial isolates were grown in YEP broth medium at 28°C for 24 h. A hundred microliters aliquots of diluted cultures (1:100) were dispensed into a 96-well plate with five replicates per isolate and incubated at 28°C for 24 h. A dye solution (crystal violet, 0/1% w/v) was used to detect biofilm production. Ten microliters of this dye solution were added into each well and left to incubate for 15 min at room temperature. The wells were then rinsed thoroughly with distilled water to wash off the unattached planktonic cells and residual dye solutions. The stained dye of biofilm cells was solubilized in 95% (v/v) ethanol, and the absorbance value was measured at 600 nm wavelength using a FLUOstar Omega Multi-Mode Microplate Reader Spectrophotometer (BMG LABTECH). Wells containing uninoculated medium served as controls and were used to determine the background absorbance. Five replicates were used per bacterial isolate.

#### Cell Wall Degrading Enzyme Activities

##### Chitinase

Chitinase production was determined by inoculation of the bacterial strains on colloidal chitin agar comprising the following (L^−1^): K_2_HPO4 (0.7 g), KH_2_PO4 (0.3 g), MgSO_4_⋅5H_2_O (0.5 g), FeSO_4_⋅7H2O (1 mg), ZnSO_4_ (1 mg), MnCl_2_ (1 mg), agar (20.0 g), and colloidal chitin (20.0 g) (Murthy and Bleakley, 2012). A total of 10 μL of overnight bacterial cultures grown in YEP broth was inoculated on the colloidal chitin agar and incubated at 28°C at dark for 5 days. Clarification zones on the agar surrounding the bacteria colony were considered as positive for chitinase synthesis. A high chitinase-producing bacterial strain, UQ284, was used as a positive control. Each isolate was tested in triplicate. Colloidal chitin was prepared with reference to Murthy and Bleakley (2012). Briefly, 10 g of chitin powder extracted from shell crab was dissolved in 75 mL of 12 M HCl with continuous stirring for 5 min, followed by 1-min stirring with 5 min intervals for one h in a fume hood. The chitin-HCl mixture was then transferred to 1 L of ice-cold distilled water and stored at 4°C overnight. The colloidal chitin was isolated by filtering the overnight mixture with two layers of coffee filter paper fitted in a Buchner funnel connected to vacuum pipe in a fume hood. Approximately 2 L of tap water at pH 8.0 was applied to rinse off the HCl attached to the colloidal chitin under the same filter assembly. The rinsing procedure completed when the pH of the filtrate reached 7.0. The moist colloidal chitin obtained was wrapped in coffee filter paper and pressed to remove moisture. Lastly, the moist colloidal chitin was autoclaved at 121°C for 20 min and stored at 4°C.

##### Protease

Protease production of the bacterial strains was assessed on a modified skim milk agar (pH 7.0) containing (L^−1^): skim milk powder (28.0 g), tryptone (5.0 g), yeast extract (2.5 g), dextrose (1.0 g), and agar (15.0 g) (Adinarayana et al., 2003). Overnight bacterial cultures grown on YEP broth were streaked with a cotton swab on agar plates and incubated at 28°C for 48 h. Clear zones surrounding the bacterial colony indicated the presence of protease. Each isolate was tested in triplicate.

##### Cellulase

Cellulase production was confirmed by growing bacterial strains on carboxymethyl cellulose (CMC) agar (pH 7.0) composed by (L^−1^): CMC disodium salt (10.0 g), K_2_HPO_4_ (1.0 g), KH_2_PO_4_ (1.0 g), MgSO_4_⋅7H_2_O (0.2 g), NH_4_NO_3_ (1.0 g), FeCl_3_⋅6H_2_O (50 mg), CaCl_2_ (20 mg), and agar (20.0 g). A total of 10 μL of overnight bacteria culture grown in YEP broth was inoculated on the CMC agar and incubated at 28°C for 4 days. After the incubation period, the agar plate was flooded with an aqueous solution of Congo red (0.5% w/v) for 15 min. The Congo red stain was then discarded and replaced with 1 M NaCl solution for 15 min. The NaCl solution was poured off, and the agar plates were observed after 10 min. The presence of clarification zones indicated a positive result for production of cellulase. A cellulolytic bacterial strain UQ7 was used as a positive control. Each isolate was tested in triplicate.

### *In planta* Assay on Lettuce

#### Measurement of Seed Germination Percentages and Vigor

Lettuce seeds (Variety Green Cos, Mr Fothergill’s, Australia) were surface-sterilized in 10% hydrogen peroxide for 30 min using the method described by Hou et al. (2012). The seeds were rinsed five times with sterile distilled water. The candidate growth-promoting bacterial isolates were pre-grown in YEP medium for 24 h at 28°C with shaking. The pre-cultures were centrifuged, and pellets were resuspended in PBS to an OD_600nm_ of 0.1 (corresponds to a concentration of 10^8^ CFU mL^−1^ of medium). The sterilized seeds were soaked in the diluted bacterial culture for 1 h at room temperature. Six lettuce seeds were then transferred to 9-cm Petri dishes containing sterile moistened filter paper. All isolates were tested using five biological replicates. Each biological replicate comprised of 18 plants (three plates). Seeds that were not inoculated with bacterial cultures and treated with only PBS were used as controls. Plates were incubated for 4 days at 26°C under dark conditions. Germination percentages (%) and root length (cm) were measured at 24 and 48 h after sterilizing seeds. The seed vigor index was measured as: Vigor index = (mean root length) × (germination percentage) (Ojha and Agrawal, 2017). The results of the measurements were analyzed statistically with one-way analysis of variance (ANOVA) and further analyzed with Tukey’s HSD Test using JMP software (SAS Institute, Cary, NC, United States).

#### Measurement of Plant Phenotypes

The bacterial isolates were also evaluated for plant growth promotion in lettuce. The selected three bacterial isolates were pre-grown in YEP medium for 24 h at 28°C in a shaker incubator. Then the isolates were diluted in PBS to an OD_600nm_ of 0.1, which corresponds to a concentration of 10^8^ CFU mL^−1^. Sterilized lettuce seeds were inoculated by submerging in a PBS suspension of a bacterial culture for 1 h. Non-inoculated seeds and those treated only with PBS were used as negative controls. After treatment, seeds were placed into a tray of fertilized UC potting mix. A hundred plants were used per bacterial treatment. Distilled water was sprayed uniformly into the soil to maintain moisture. The trays were incubated in the growth cabinet (short day, 8 h light) at 26°C for 14 days. Root and shoot lengths were measured and recorded. The results of the measurements were analyzed statistically with one-way analysis of variance (ANOVA) and further analyzed with Tukey’s HSD Test using JMP software (SAS Institute, Cary, NC, United States).

#### Inoculation of Bacterial Isolates Into Chili Plants

The bacterial suspensions were prepared as follows: each bacterial inoculum (prepared from a single colony obtained from an overnight culture streaked on the plate) was added to YEP broth and incubated for 12 h at 28°C with shaking at 160 rpm. After centrifugation, the supernatant was discarded, and the pellet washed at least once and resuspended in PBS at a final cell density of 10^8^ CFU mL^−1^ (OD_600nm_ of 0.1). The inoculation of chili pepper with the bacterial suspension was done twice; the first application was before cultivation when seeds were soaked for 1 h in the bacterial suspension. The second application was carried out on the soil surface adjacent to each seedling 3 weeks after planting, prior to pathogen inoculation. *P. capsici* culture was maintained on clarified V8 (cV8) agar at room temperature under dark conditions. *P. capsici* disks (5 mm diameter) of 6-day-old culture were placed at the center of cV8 plates and incubated at 28°C overnight. The pathogen inoculum was prepared by adding organic wheat grains (100 g) and water (72 mL) into a 500 mL Erlenmeyer flask. The flask was capped with aluminum foil and autoclaved twice consecutively. The flask was then shaken to homogenize the mixture, and the seeds were inoculated with four seven mm-diameter agar plugs from a *P. capsici* culture that was actively growing in the cV8 medium. The inoculated wheat seeds were incubated at room temperature (21 ± 2°C) under constant fluorescent light for 4 weeks. The *Phytophthora*-infested wheat grains were then placed at the bottom of the pot by uprooting the plant. For maintaining water-saturated conditions, the trays containing the plants were placed in clear plastic trays without holes and filled with water. After 72 h the water was drained, the clear plastic tray removed and then the soil was allowed to dry naturally, followed by regular daily watering. The effect of the tested microbes on plant growth was measured by monitoring weekly the overall plant phenotype, height and leaf number for a month.

### Chili Plant Protection Assay Against *P. capsici*

#### *P. capsici*-Infected Chili Plant Cultivation

*In vivo* experiments were conducted to assess the efficacy of the bacterial isolates in suppressing pathogen load of the causal agent of *Phytophthora* blight in chili pepper (*C. annuum* L., cultivar Cayenne). Seeds were surface-sterilized with 70% (v/v) ethanol for 5 min, then with 5% sodium hypochlorite for further 5 min and rinsed three times with sterile distilled water. The sterilized seeds were soaked in the bacterial suspension containing 10^8^ CFU mL^−1^ (OD_600nm_ of 0.1) for 1 h. Each seed was planted in an 80 mm-square pot filled with the UQ23 potting mix (70% composted pine bark 0–5 mm, 30% cocoa peat, mineral fertilizer) and arranged in a randomized block design. One seedling was used per pot, and each treatment consisted of five replicates. Two control treatments were included; one was included plants infected with pathogen only (C1) and the other was not inoculated with the pathogen or any bacterial isolate but treated with only PBS instead (C2). The plants were evaluated for shoot health, plant height. Root rot and leaf disease severity were assessed using a scale of 0–5: 0 = no visible disease symptoms and normal appearing plant; 1–4 = plants with increasing levels of leaf wilting, dropping and curling or roots with increasing levels of root rot; 5 = plants with severe symptoms.

#### Measurement of Photosynthetic Activity of *P. capsici*-Infected Chili Plants

The net assimilation rate of CO_2_, photosynthetic and transpiration rates, as well as stomatal conductance, were measured from individual leaves of each plant sample with a closed photosynthesis system an LI-6400XT Portable Photosynthesis System (LI-COR, NE, United States). To determine the effect of the disease on the CO_2_ rate, photosynthetic and transpiration rates as well as stomatal conductance, completely randomized design was used with ten replicates (10 plants) growing in a growth cabinet (short photoperiod SD; 8 h light: 16 h dark at 28°C during the day and 20°C at night). The pathogen inoculation method was described in Section “Inoculation of Bacterial Isolates Into Chili Plants.” Measurements were taken 2 weeks after the pathogen inoculation.

#### Measurement of *P. capsici* Load on Chili Roots via Quantitative PCR

Five chili pepper roots (100 mg for each sample) which included the entire infected area with necrotic lesions were collected in a 50 mL Falcon tube. Plant tissue was ground and homogenized using a mortar and pestle filled with liquid nitrogen. DNA was then extracted from ground tissue using the ISOLATE II Plant DNA kit (Bioline Inc., Taunton, MA, United States) following the manufacturer’s instructions (support protocol for purifying fungal/oomycete genomic DNA). Just before the extractions were initiated, one nanogram of a plasmid containing an exogenous gene to the soil (green fluorescent protein, GFP) was added to each tube immediately after the first extraction buffer. This allowed to account for DNA extraction efficiencies.

Real-time quantitative PCR (qPCR) assays to quantify the amount of pathogen DNA in different treatments were conducted using *P. capsici* specific primers based on internal-transcribed spacer (ITS) sequence, Pcap-q-1-F (5′-GGA ACC GTA TCA ACC CTT TTA GTT G-3′) and Pcap-q-1-R (5′-CGC CCG GAC CGA AGT C-3′) and a housekeeping gene was used as a plant reference gene using Ca_pAO_Fw_59 (5′-GGGCCCCGTTGGAATG-3′) and the reverse primer Ca_pAO_Rv_59 (5′-TGGGACTTGATTGCAACACTCT-3′), as described by Garcıì et al. (2004). The GFP primers used as a spike for measuring DNA extraction efficiency were GFP5_qrt_F (5′-CCAGACAACCATTACCTGTCC-3′) and GFP5_qrt_R (5′-CCATGTGTAATCCCAGCAGC-3′). *P. capsici* primers were designed with the Primer Express software version 3.0.1 (Applied Biosystems).

Quantitative PCR (qPCR) was performed using the using QuantStudio 12K Flex Real-Time PCR System (Applied Biosystems) apparatus, and results were analyzed with the manufacturer’s software (QuantStudio^TM^ Real-Time PCR Software v1.1). Each reaction mixture (10 μL) contained 4 μL of DNA, SYBR green 5 μL master mix, and 0.5 μM of each primer. All DNA samples were standardized to the concentration of the lowest sample, and 4 μL was added to the reaction. No-template control reactions contained the same reagent concentrations, but 4 μL of ultra-pure DNA-free water was added instead of a DNA template. The thermal cycling conditions consisted of an initial denaturation step at 95°C for 2 min followed by 40 cycles at 95°C for 30 s, 56°C for 30 s, and 72°C for 1 min. A final extension step at 72°C for 5 min was added. A standard curve was generated by plotting the cycle numbers of a pathogen DNA dilution series (in the *Y*-axis) against the known concentration of *P. capsici* DNA (*X*-axis) (see **Supplementary Figure S1**). The PCR efficiency for the standard curves for both the primer sets was *R*^2^ = 0.99. Starting from 400 ng of DNA, dilutions were done in six steps with 10-fold dilutions per step (0.0001–10 ng). A linear correlation (*R*^2^ = 0.9963) was obtained between the *C*t values and the *P. capsici* DNA concentration. The efficiency of the amplifications for *P. capsici* and plasmid primers was 100% (slope = −3.316). We calculated the relative *P. capsici* DNA biomass normalized to GFP plasmid by using the following formula (Gao et al., 2004):

$$\begin{array}{l} \text{Relative}\;\text{C}\text{t value of}\;\text{P. capsici}\text{,}\Delta \text{C}{\text{t}}_{\text{P}.\text{capsici}}\\ =Ct_{\text{P}.\text{capsici}}--Ct_{\text{GFPplasmid}} \end{array}$$

$$\begin{array}{l} \text{Relative }\text{P. capsici}\text{ biomass,}\Delta \text{P. capsici}\text{ pg }=\;\text{antilog}\\ \left[(\Delta Ct_{\text{P}.\text{capsici}}--22.363)/(-3.316)\right] \end{array}$$

### Extraction and Identification of Potential Anti-oomycete Compounds

Each bacterial strain was grown in 50 mL YEP liquid cultures until stationary phase. The culture was then extracted with an equal volume of ethyl acetate (Fisher Scientific, NJ, United States). The organic layer was collected and evaporated to dryness at 40°C *in vacuo* (Rotavapor, Büchi, Switzerland). The residue was then successively triturated with three different solvents of different polarity: hexane, dichloromethane, and ethyl acetate, respectively. Compounds in the extracts were identified using a Shimadzu (Kyoto, Japan) GCMS-QP2010ULTRA gas chromatography-mass spectrophotometer equipped with a Restek (Bellefonte, PA, United States) Rtx-5MS (Crossbond diphenyl dimethyl polysiloxane) capillary column (30 m × 0.25 mm × 0.25 μm). Samples (1 μL) were injected in the split mode with a split ratio of 10. The injector temperature and initial oven temperature were 240 and 160°C, respectively. Helium was used as the carrier gas at a constant linear velocity of 46.6 cm s^−1^. The oven temperature program was as follows: isothermal 160°C, 1 min; temperature gradient 160–300°C at 10°C min^−1^. The mass spectrometer was operated with an ion source temperature of 200°C and interface temperature of 300°C. The analysis was done in a full-scan mode with a mass range of 42–500 m/z. The peaks were finally identified by comparison to National Institute of Standards and Technology (NIST) 14 library with similarity indices (SI) >80.

## Results

### Identification of Bacterial Isolates by 16S rDNA Amplicon Sequencing

Soil bacterial isolates were identified by near-full length 16S rDNA gene amplification and sequencing. The presence and length of the amplicons were confirmed on a 1% agarose gel (**Supplementary Figure S2**). Forty-eight isolates were successfully sequenced and identified by BLAST matching. Of the seven identified genera, *Pseudomonas* was the most prevalent (59% of samples). Sequences were deposited into the NCBI GenBank database under accessions given in **Supplementary Table S1**. The highest hits with 99% similarity obtained after blasting the sequence reads against the National Center for Biotechnology Information database^1^ are shown in **Supplementary Table S1**.

### Screening and Identification of Bacterial Isolates for Antagonistic Activity

**FIGURE 1:**
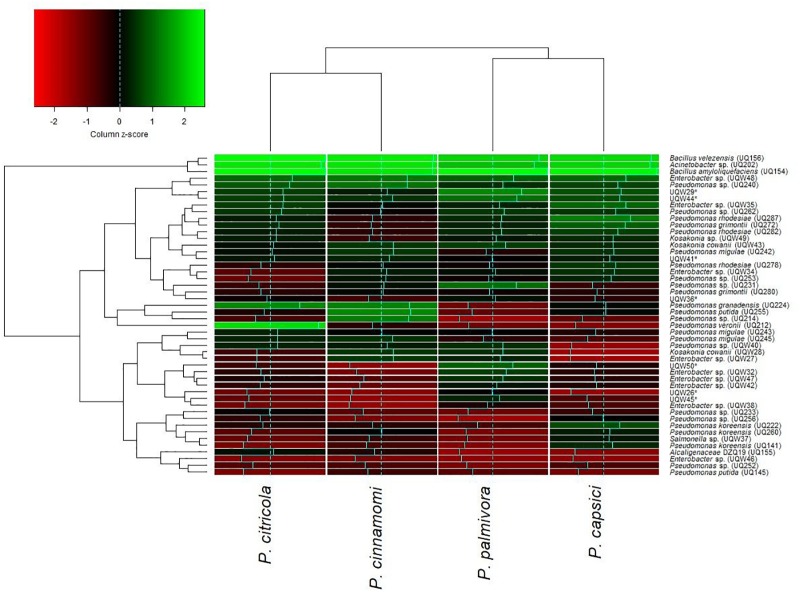
Hierarchically clustered heatmap of relative activity of 48 putative soil bacterial isolates against four species of *Phytophthora* with economic importance. Dendrograms were defined using the complete-linkage method and z-scores were derived using column scaling. The intensity of the green color indicates high levels of inhibition and the intensity of the red color indicates low levels of inhibition.

**FIGURE 2:**
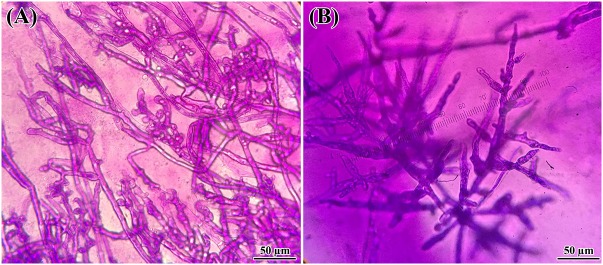
Changes in hyphal morphology of *P. citricola* co-inoculated with *B. velezensis* (UQ156) on PDA. Morphological alterations were observed in hyphae during the interaction of **(A)** *P. citricola* and isolate *B. velezensis* (UQ156). Excessive branching and irregular shapes were observed in the presence of the **(A)** bacterium **(B)** single culture of *P. citricola*.

One of the objectives of this study was to screen for potential isolates that exhibit plant growth promoting and biocontrol properties, using various existing biochemical microbiological *in vitro* assays. Bacteria isolated from *Arabidopsis* rhizosphere and bulk soil were investigated for plant growth promotion and antagonistic activities against pathogenic *Phytophthora* spp. isolates. Overall, the isolates showed varied levels of inhibition against *Phytophthora*. Almost all 48 tested isolates showed inhibitory effects against *Phytophthora* (**Figure 1**). These results indicated that isolates grown on PDA plates released an extracellular diffusible metabolite(s) that inhibited the growth of *Phytophthora* spp. The strongest activity was observed for *Bacillus amyloliquefaciens* (UQ154), *Bacillus velezensis* (UQ156), and *Acinetobacter* sp. (UQ202) that were chosen for analyses that are more detailed. Microscopic observation of *Phytophthora* hyphae showed an abnormal morphology, i.e., excessive branching and irregular shape along with the inhibition zone in dual cultures plate assay (**Figure 2**). This was found for *B. amyloliquefaciens* (UQ154), *B. velezensis* (UQ156), and *Acinetobacter* sp. (UQ202) but not for the other isolates tested.

### Plant Growth Promoting Traits

#### Nitrogen Fixation

Nitrogen-fixing ability was determined by culturing the isolates in a nitrogen-free bromothymol blue (NfB) medium. Nitrogen-fixing isolates increased the pH of the medium, which is determined by the color change of the medium from green to blue (Bhattacharyya and Jha, 2012). *B. amyloliquefaciens* (UQ154) and *B. velezensis* (UQ156) are nitrogen-fixing bacteria since the medium color changed to blue (**Supplementary Figure S3**). Furthermore, signs of bacterial growth were observed in a radial pattern as highlighted by the red circles. In contrast, the medium color for *Acinetobacter* sp. (UQ202) remained unchanged, and no growth of bacteria was observed indicating that *Acinetobacter* sp. (UQ202) is not a nitrogen-fixing bacterium.

#### Phosphate Solubilization

All isolates were found to solubilize phosphate with similar efficiencies. All isolates produced distinct halos (clear zone > 1 mm) around the colony on the plate. Colony diameter, halo zone diameter, and solubilization index of the isolates are presented in **Table 1**. No statistically significant differences were observed for Pi solubilizing indices.

**Table 1:** Phosphate solubilization of bacterial isolates in plate assays.

Bacterial isolate	Colony diameter (mm)	Halo zone diameter (mm)	Pi solubilizing index (PSI)^a^
UQ154	6.67 ± 1.35^ab^	8.00 ± 1.73^a^	2.20 ± 6.12^a^
UQ156	6.33 ± 2.19^ab^	7.67 ± 0.98^a^	2.21 ± 4.32^a^
UQ202	4.67 ± 2.75^b^	6.33 ± 2.09^b^	2.36 ± 3.62^a^

*Means within a column followed by the same letter are not significantly different at P < 0.05.*

#### Production of Siderophores

All strains induced a change in color in Chromeazurol agar (see **Supplementary Figure S4**) which indicates that they can produce siderophores. A positive siderophore reaction by the CAS method shows a yellow halo surrounding the bacterial colonies grown under iron-limiting conditions (Schwyn and Neilands, 1987). This is the universal assay developed so far for siderophores; it only depends on the ability of the compound to bind iron with relatively high affinity. All strains of were able to produce catechol-type and hydroxamate-type siderophores to various degrees, as shown by the Arnow assay (Arnow, 1937) and Csáky assay (Csáky, 1948), respectively (**Table 2**).

**Table 2:** Siderophores production by the bacterial isolates.

Strain	CAS-agar universal test	Csáky test (hydroxamate-type)	Arnow test (catechol-type)
UQ154	++	+	++
UQ156	+++	+	+
UQ202	++	+	+

*The symbols represent the relationship between the halo average diameter and the average diameter of the colony growth (+: small; ++: medium; +++: big) for CAS-Agar universal test, and the intensity of production of siderophores (−: none; +: low; ++: high; +++: very high) for Csáky and Arnow tests.*

#### IAA Production

**FIGURE 3:**
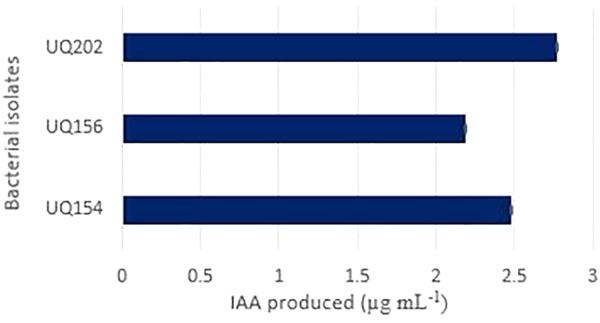
Indole acetic acid (IAA) produced by the isolates *Acinetobacter* sp. (UQ202), *B. velezensis* (UQ156), and *B. amyloliquefaciens* (UQ154) in presence of L-tryptophan in the growth medium.

It was observed that all strains could produce IAA (**Figure 3**) when L-tryptophan was supplemented in the medium. The culture supernatant changed to red color after the addition of the Salkowski’s reagent.

#### Biofilm Production

**FIGURE 4:**
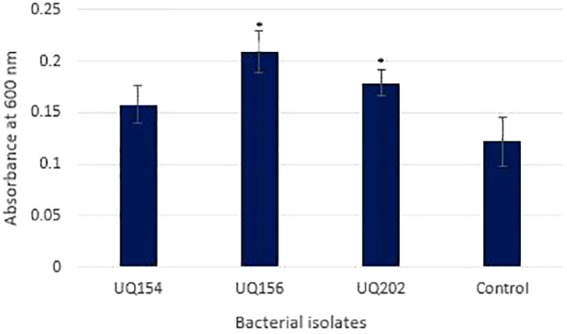
Biofilm production assay of bacterial isolates using 96-well microtiter plates after 24 h. Asterisks (^∗^) represent data that are significantly (P < 0.05) different compared to the negative control (medium only).

Biofilm production results showed that *B. velezensis* (UQ156) and *Acinetobacter* sp. (UQ202) had significantly higher absorbance values than the negative control, which suggests that these isolates can produce biofilms (**Figure 4**). In contrast, *B. amyloliquefaciens* (UQ154) did not have this attribute.

#### Cell Wall-Degrading Enzyme Activities

**FIGURE 5:**
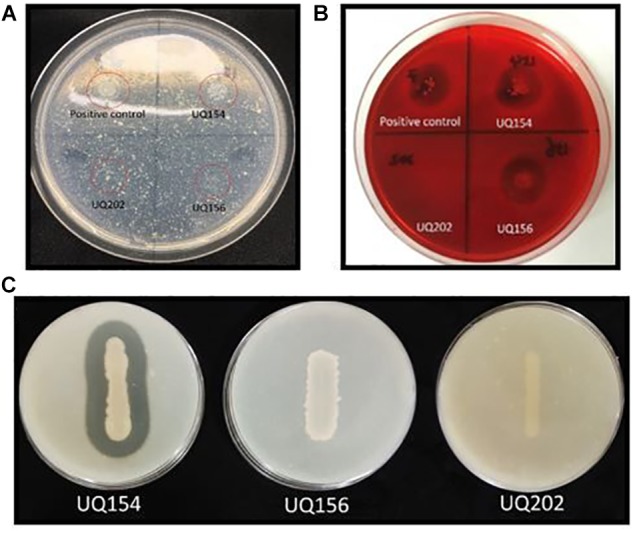
**(A)** The red-circled isolates showed isolates grown on colloidal chitin agar, **(B)** Clear zone around the colonies grown on CMC agar plate after staining, indicate the production of cellulases, and **(C)** Production of clear zone around the isolate indicates the production of proteases.

The isolates were tested for their ability to produce cell wall-degrading enzymes. Compared with the positive control, no clarification zones were observed for *B. amyloliquefaciens* (UQ154), *B. velezensis* (UQ156), and *Acinetobacter* sp. (UQ202), indicating that none of these three bacterial strains synthesized chitinase (**Figure 5A**). However, clarification zones indicative of cellulose production were present in *B. amyloliquefaciens* (UQ154) and *B. velezensis* (UQ156) (**Figure 5B**). *B. velezensis* (UQ156) and *Acinetobacter* sp. (UQ202) do not seem to produce cellulases as no clarification zones were present. A clarification zone indicative of protease production was present only in *B. amyloliquefaciens* (UQ154) (**Figure 5C**).

### All Selected Isolates Improved Seed Emergence

**FIGURE 6:**
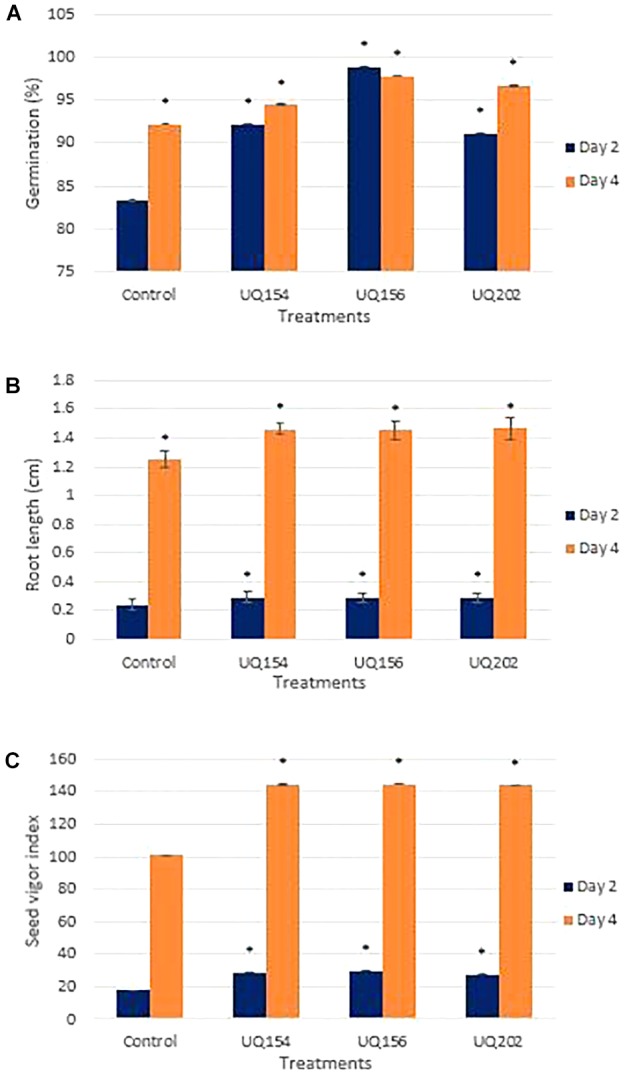
Effect of bacterial inoculation on lettuce seedling germination percentage **(A)**, root length **(B)**, and seed vigor index **(C)** after inoculation with bacterial isolates after 2 and 4 days of germination. For each panel, asterisks (^∗^) represent data that are significantly different compared to the respective controls (P < 0.05).

Seedling germination assays showed that all isolates enhanced germination percentages and seedling root growth in lettuce. Increases ranged from 9.33 to 18.67% in germination percentages and between 16.0 and 16.8% in root length of lettuce seedlings (**Figures 6A,B**). Seed inoculation also contributed to better seed vigor (**Figure 6C**). Lettuce seeds individually inoculated with bacterial isolates exhibited a significant increase in seed vigor index after 4 days, compared to the control.

#### All Bacterial Isolates Promoted Lettuce Growth

**FIGURE 7:**
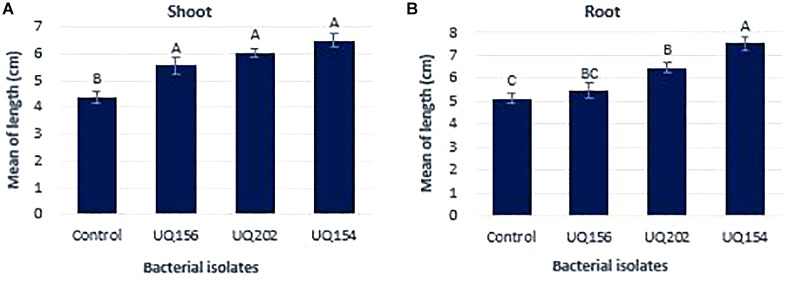
Effect of bacterial inoculation on lettuce shoot weight **(A)** and root length **(B)** after 14 days of growth. Different letters above bars indicate a statistically significant difference (P < 0.05).

All bacterial isolates contributed to a significant increase in shoot length of lettuce seedlings, with the highest increase being 32.26% from the plant inoculated with *B. amyloliquefaciens* (UQ154) (**Figure 7A**) in lettuce growth promotion assay. Only *Acinetobacter* sp. (UQ202) and *B. amyloliquefaciens* (UQ154) stimulated root growth with the latter promoting the highest root length (**Figure 7B**).

### Biocontrol Activity of Bacterial Isolates on *P. capsici*-Infected Chili-Pepper Plants

#### Photosynthetic Parameters

**FIGURE 8:**
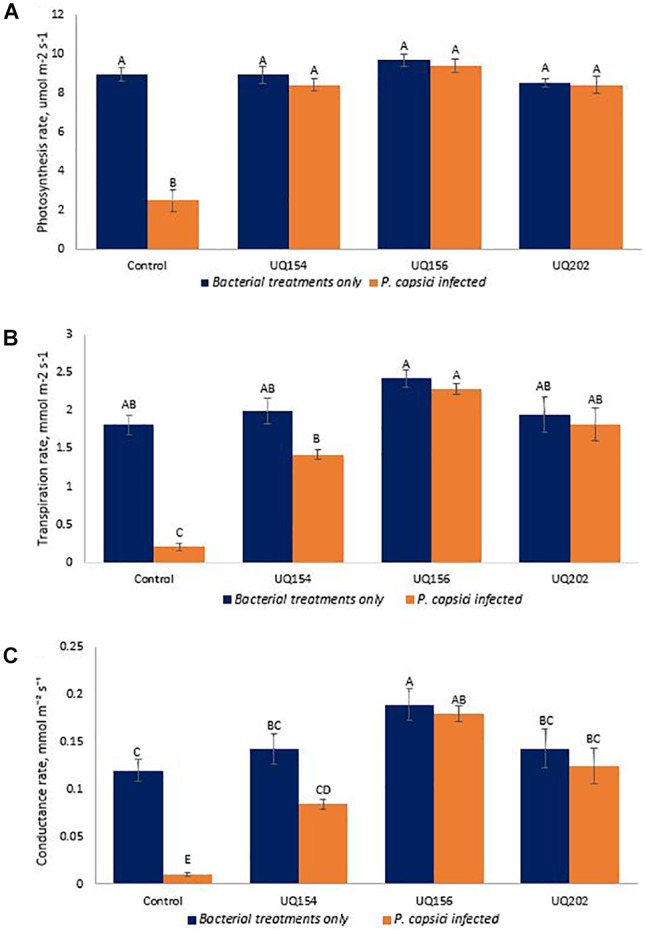
Photosynthetic carbon assimilation **(A)**, leaf transpiration **(B)**, and stomatal conductance **(C)** of chili pepper infected with *P. capsici* inoculated with bacterial isolates. Different letters above bars indicate a statistically significant difference (P < 0.05). ^∗^PC is referred to *P. capsici*.

Photosynthetic parameters of *Phytophthora*-inoculated chili plants inoculated with bacterial isolates were measured using an LI-COR LI-6400XT Portable Photosynthesis System to profile carbon assimilation, leaf transpiration, and stomatal conductance. The isolates were screened simultaneously by seedling assays in pot trials during the three-way interaction of pathogen, bacterial antagonists and host plant to mimic field-like conditions, where *P. capsici* also infects the plants through the roots. Inoculation with all isolates increased photosynthetic carbon assimilation, leaf transpiration, and stomatal conductance in *P. capsici*-infected plants (**Figure 8**). However, no differences were found between healthy plants inoculated with each of the isolates and the uninfected control.

#### Bacterial Inoculation Reduced Pathogen Load of *P. capsici*-Infected Chili

**FIGURE 9:**
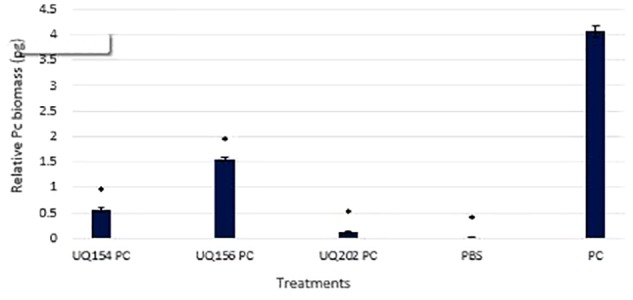
Detection and quantification of *P. capsici* DNA in chili plant roots using qPCR. Asterisks (^∗^) indicate statistically significant differences compared to the *Phytophthora*-infected control.

To measure pathogen load after bacterial inoculations, *P. capsici* DNA was quantified by qPCR. Measurement of pathogen biomass relative to the amount of plant host DNA was avoided as this may lead to an overestimation of pathogen biomass quantified, especially toward the late stages of infection, where plant DNA degradation is often linked to plant cell death (Brouwer et al., 2003; Eshraghi et al., 2011). Similar methods have been used for quantifying human pathogens (Damen et al., 2008; Halliday et al., 2010). Bacterial inoculation led to lower pathogen loads in chili (*P* ≤ 0.0001). The sensitivity of the qPCR to detect small amounts of *P. capsici* DNA in the samples was observed (<0.001 ng). This is consistent with other studies that reported the ability to detect small amounts of *Phytophthora* DNA in plant tissues (Vandemark and Barker, 2003; Silvar et al., 2005). The results of the relative *P. capsici* DNA biomass in infected chili roots was 4.06 pg in the uninoculated control (**Figure 9**).

However, upon inoculation with *B. amyloliquefaciens* (UQ154), *B. velezensis* (UQ156), and *Acinetobacter* sp. (UQ202), the relative pathogen DNA biomass was significantly lower (0.56, 1.56, and 0.12 pg, respectively). The efficiency of bacterial antagonists on the control of *P. capsici* of chili pepper was evaluated (**Table 3**). Because the primary pathogen is soil-borne that persists in the soil; infection often starts from the roots and lower part of the stem, followed by a sudden wilting of foliage and leaves and eventually resulting in the death of the plants (Babadoost, 2005). In the *P. capsici* inoculated control, much more pronounced symptoms of the disease were observed, including wilting and dropping in the upper part of plants, whereas the plants treated with antagonistic bacterial strains showed a markedly reduced appearance of the symptoms. All the strains showed a similar reduction of disease severity on chili pepper plants (**Table 3**). We noticed higher root biomass in plants inoculated with bacterial isolates compared to uninoculated, uninfected or *P. capsici*-infected chili plants (**Supplementary Figure S5**).

**Table 3:** Effect of antagonistic bacterial isolates against *P. capsici* disease development on chili pepper plants.

Treatment	Disease severity	Plant height (mm)	Root length (mm)
UQ154	3.0 ± 0.0	86.7 ± 0.43	18.7 ± 1.81
UQ156	3.0 ± 0.0	88.2 ± 0.64	19.5 ± 2.3
UQ202	2.0 ± 0.0	95.2 ± 0.35	21.2 ± 1.3
Control (PBS)	−	87.4 ± 0.53	19.8 ± 3.1
*P. capsici*	5.0 ± 0.0	84.2 ± 0.4	14.9 ± 2.2

*Disease severity was assessed on a 0 to 5 scale from 0 = no visible disease symptoms to 5 = severe symptoms. Results are an average of five replicates. Values are mean ± standard error.*

#### Isolation of Putative Anti-oomycete Bacterial Compounds

**FIGURE 10:**
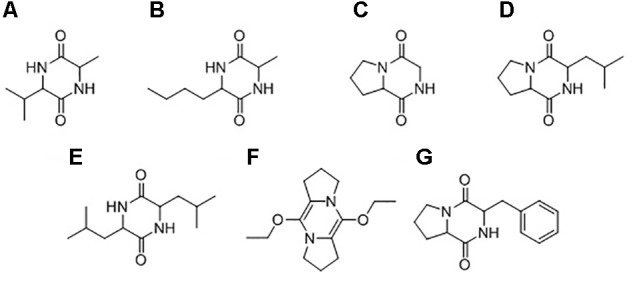
Chemical structures of compounds identified in the bacterial extracts: **(A)** 3-isopropyl-6-methylpiperazine-2,5-dione, **(B)** 3-butyl-6-methylpiperazine-2,5-dione, **(C)** hexahydropyrrolo[1,2-a]pyrazine-1,4-dione, **(D)** 3-isobutylhexahydropyrrolo[1,2-a]pyrazine-1,4-dione, **(E)** 3,6-diisobutylpiperazine-2,5-dione, **(F)** 5,10-diethoxy-2,3,7,8-tetrahydro-1H,6H-dipyrrolo[1,2-a:1′,2′-d]pyrazine, **(G)** 3-benzylhexahydropyrrolo[1,2-a]pyrazine-1,4-dione.

**FIGURE 11:**
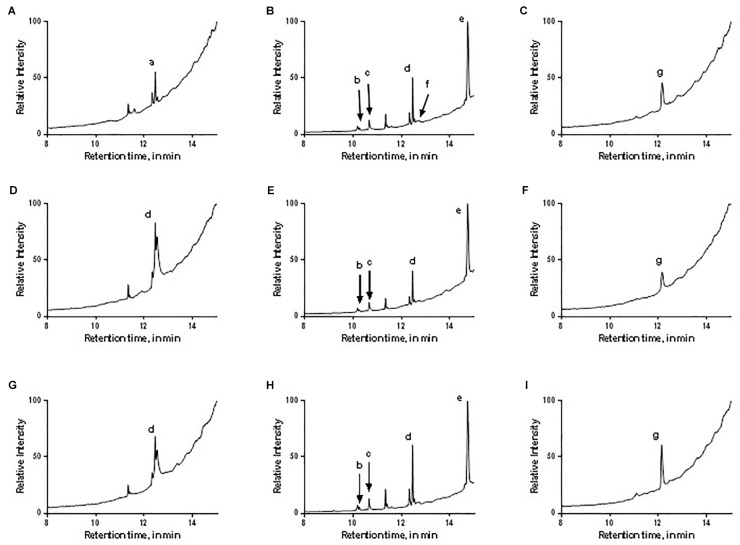
Total ion chromatograms of the different bacterial extracts: **(A–C)** B. amyloliquefaciens (UQ154), **(D–F)** B. velezensis (UQ156), **(G–I)** Acinetobacter sp. (UQ202) hexane, dichloromethane, and ethyl acetate extracts, respectively. The letters correspond to the identity of the peaks as labeled in **Figure 10**.

GC-MS was used to characterize the potential anti-oomycete compounds extracted in hexane, dichloromethane, and ethyl acetate. **Figure 10** lists the structures of the chemical compounds found in the extracts and **Figure 11** shows the total ion chromatograms of all the bacterial extracts. The dichloromethane extracts have more peaks than the other two extracts, indicating that most of the extracted compounds are polar. Using the NIST14 library, most of the peaks were identified to be DKPs. For the hexane extracts (**Figures 11A,D,G**), only one peak with SI > 80 was detected and identified to be 3-isopropyl-6-methylpiperazine-2,5-dione for *B. amyloliquefaciens* (UQ154), while it was identified as 3-isobutylhexahydropyrrolo[1,2-a]pyrazine-1,4-dione in the other two isolates. For the dichloromethane extracts (**Figures 11B,E,H**), five peaks with SI > 80 were detected for *B. amyloliquefaciens* (UQ154) and identified to be 3-butyl-6-methylpiperazine-2,5-dione, hexahydropyrrolo[1,2-a]pyrazine-1,4-dione, 3-isobutylhexahydropyrrolo[1,2-a]pyrazine-1,4-dione, 3,6-diisobutylpiperazine-2,5-dione, and 5,10-diethoxy-2,3,7,8-tetrahydro-1H,6H-dipyrrolo[1,2-a:1′,2′-d]pyrazine. In contrast, one compound, 5,10-diethoxy-2,3,7,8-tetrahydro-1H,6H-dipyrrolo[1,2-a:1′,2′-d]pyrazine, were not found in *B. velezensis* (UQ156) and *Acinetobacter* sp. (UQ202). Finally, for the ethyl acetate extracts (**Figures 11C,F,I**), only one peak with SI > 80 was detected in all isolates and identified to be 3-benzylhexahydropyrrolo[1,2-a]pyrazine-1,4-dione.

## Discussion

Rhizosphere soil and roots play an important role in recruiting and maintaining bacterial antagonists (Venturi and Keel, 2016). Root-associated bacteria often display plant beneficial attributes such as plant growth promotion and the capability of suppressing plant pathogens acting as biocontrol agents (Naureen et al., 2017; Thirumal et al., 2017). In this study, a total of 48 isolates (**Figure 1**) was screened by dual culture plate assays for *in vitro* antagonism against the soil-borne pathogen *Phytophthora* spp. Three isolates belonging to the genera *Bacillus* and *Acinetobacter* were found to control *Phytophthora* spp. *in vitro* and *in vivo*. *B. amyloliquefaciens* and *B. velezensis* have been widely reported as plant growth promoters and biocontrol agents against a broad range of soil-borne pathogens including Fusarium wilt of banana and potato dry rot caused by *Fusarium* sp. (Recep et al., 2009; Wang et al., 2013; Xu et al., 2013; Meng et al., 2016; Adibi et al., 2017). A recent study showed that *B. velezensis* had a broad-spectrum biocontrol activity via antagonism against two foliar bacterial pathogens and soil-borne fungal pathogens (Liu et al., 2017). *Acinetobacter* sp. were also reported as the potential biocontrol agent against fungal diseases and plant-growth promoter (Liu et al., 2007; Rokhbakhsh-Zamin et al., 2011).

### Biocontrol and Plant Growth Promotion Traits

Mechanisms of plant growth promotion can be categorized into two: direct and indirect. Direct mechanisms include nitrogen fixation, phosphate solubilization, root colonization (biofilm production), IAA production while indirect mechanisms include cell wall degrading enzyme production, and antibiosis (Ngoma et al., 2012; Shaikh et al., 2016). The production of siderophores can be considered as both direct and indirect (Shaikh et al., 2016).

The isolates exhibited potent inhibitory effects toward *P. capsici, P. citricola, P. cinnamomi*, and *P. palmivora in vitro*. Microscopic observation of *Phytophthora* hyphae showed an abnormal morphology, i.e., excessive branching and irregular shape along with the inhibition zone in dual-culture plate assays. Distinct morphological alterations in *P. citricola* hyphae were also observed under light microscopy in dual-culture assays with the soil bacterial isolate *B. velezensis* (UQ156). Compared to the control plates with *Phytophthora* only, the hyphal features of the *Phytophthora* culture observed in the presence of bacterial colonies displayed excessive branching and irregular shapes (**Figure 2**). These morphological abnormalities in *Phytophthora* pathogens could be related to interference with membranes or the production of lytic and cell wall degrading enzymes, mainly cellulase, chitinase, antibiotics, and other secondary metabolites by antagonistic bacteria as reported in recent studies (Bednarek, 2012; Miedes et al., 2014; Nagpure et al., 2014). Gupta et al. (2001) also observed hyphal perforations, lysis, and fragmentation in *Macrophomina phaseolina* and *Sclerotinia sclerotiorum* in dual culture assay with *Pseudomonas fluorescens* isolated from the rhizosphere of potato. A similar finding was reported in a biocontrol study of multiple plant diseases by plant growth-promoting rhizobacteria (PGPR) (Liu et al., 2017). However, bacterial secondary metabolite production is highly dependent on the cultivation media and conditions as well as the inoculum quantity/concentration (Blom et al., 2011). We tested the isolates for chitinase, cellulase, and protease production. None of the isolates showed positive activity in chitinase production on colloidal chitin agar (**Figure 5A**). *B. amyloliquefaciens* (UQ154) and *B. velezensis* (UQ156) were found to produce cellulase enzyme to break down cellulose in CMC agar (**Figure 5B**). This is consistent with an apparent reduced cell wall thickness for isolate *B. velezensis* (UQ156) that requires further verification. Additionally, *B. amyloliquefaciens* (UQ154) was also found positive in the production of protease on a modified skim milk agar (**Figure 5C**). Interestingly, *Acinetobacter* sp. (UQ202) did not prove to produce any of the hydrolytic enzymes indicating that its biocontrol activity could be due to other mechanisms.

The isolates were CAS-positive for siderophores production and were able to produce catechol-type and hydroxamate-type siderophores to various degrees. The ability of the bacterial isolates to produce siderophores may be involved in the antagonistic activity observed in this study as they can deprive iron of other competing organisms (Mata et al., 2001; Deacon, 2013). Iron is one of the fundamental growth elements needed by all living organisms (Braun and Hantke, 2011). The insufficiency of iron in soil environments and on plant surfaces often leads to a competition between organisms (Traxler et al., 2012). Therefore, siderophores may assist the biocontrol activity by confiscating iron from pathogens, hence limiting their growth (El-Tarabily and Sivasithamparam, 2006). Bacteria that produce high affinity and specific siderophores that can be utilized by the producer but not by other microbes could be potentially used as biological control agents (Thomashow and Bakker, 2015).

Apart from their role in the active transport of iron (III), siderophores may act as growth factors, and some are potent antibiotics (Leong, 1986; Dwivedi and Johri, 2003; Miller et al., 2009). Production of these iron-binding agents by the beneficial bacteria also stimulate plant growth in the presence of other potentially harmful rhizosphere microorganisms (Souza et al., 2015; Olanrewaju et al., 2017). These harmful rhizosphere microorganisms may affect plant growth by interfering with the transport of plant growth metabolites or nutrients to plants (Schippers et al., 1987). Campbell et al. (1986) observed a reduction in emergence of canola seeds inoculated with *Pseudomonas* isolate rp2 in the field by 30–40% (Campbell et al., 1986). In another study, Kennedy et al. (1991) investigated an isolate of *P. fluorescens* which was highly inhibitory to downy brome (*Bromus tectorum* L.) *in vitro* and in the field (Kennedy et al., 1991).

Another important growth promoting traits of beneficial bacteria are the abilities to fix nitrogen and solubilize phosphate. These traits increase the available nutrients in the soil, which the plant can absorb for growth (Vessey, 2003). Nitrogen-fixing bacteria can convert N_2_ from the air into ammonium, which is easily absorbed by plants. Two of the three isolates showed nitrogen fixation capability (**Supplementary Figure S3**). This result could explain the plant growth promotion effect of *B. amyloliquefaciens* (UQ154) and *B. velezensis* (UQ156). However, the same cannot be said of *Acinetobacter* sp. (UQ202). In addition, phosphorus is another important element for plant health. In fact, it is categorized as a macronutrient for plants (Bansal and Siddiqui, 2017). In addition to comprising a crucial component of nucleotides, transcriptional regulators of the phosphate starvation response have been shown to directly repress defense responses (Castrillo et al., 2017). However, most of the phosphorus present in soil is in the form of insoluble phosphates, which cannot be utilized directly by plants (Turan et al., 2007). Hence, the ability of these bacterial isolates to solubilize phosphate is an important trait for promoting plant growth.

The production of phytohormones is another direct mechanism of PGPR. IAA production is a major attribute of most rhizosphere bacteria that stimulate plant growth. In this study, all the bacterial strains showed the ability to synthesize IAA in the presence of L-tryptophan with IAA concentrations ranging from 2.19 to 2.77 μg mL^−1^ (**Figure 3**). Changes of LB broth color supplemented with L-tryptophan was considered as a qualitative indication of the production of IAA.

Production of biofilms by beneficial bacteria associated with roots has been found to be important for plant growth, yield, and crop quality (Seneviratne et al., 2010). Enhancement of plant growth by root-colonizing *Bacillus* sp. and *Acinetobacter* sp. have been well documented (Kloepper et al., 2004; Borriss, 2011; Rokhbakhsh-Zamin et al., 2011; Meng et al., 2016). For example, *Acinetobacter* sp. has been reported for multiple broad-spectrum plant growth–promoting activity, root colonization in pea and efficient inorganic and organic phosphate-solubilization (Gulati et al., 2009). It should be noted that, while the studied isolates exhibited multiple traits considered typical of PGPR *in vitro*, these activities may not be responsible for the PGPR effects observed *in situ*. Further experiments to elucidate the specific mechanism underpinning the observed beneficial activities of these isolates would be prudent.

### Plant Growth Promotion

Results from our study indicated that treatment of lettuce seeds with the selected beneficial bacterial isolates significantly improved seed vigor and seedlings growth (**Figure 6**). Different mechanisms have been reported for similar activities in other beneficial soil bacteria strains. For instance, PGPR strains are hypothesized to enhance seed germination and vigor index by reducing the incidence of seed mycoflora which is unfavorable for plant growth (Gond et al., 2015). Han and Lee (2005) also reported an increase in the growth of lettuce inoculated with PGPR. Increases in germination, seedling vigor, plant height, leaf area, tillering capacity, seed weight have been shown to be translated to higher grain yields under glasshouse and field conditions after inoculation of millet seeds with *P. fluorescens* (Raj et al., 2004).

The ability of bacterial isolates to increase the seed emergence, vigor, and germination has previously been associated with secretion of several substances, e.g., plant growth promoting compounds, such as plant growth regulators and phytohormones produced by bacteria that enhanced various stages of plant growth (Podile and Kishore, 2007; Meng et al., 2016). Herrera et al. (2016) also observed an increase in chlorophyll content of barley seedlings inoculated with seed-derived endophytic bacterial inoculants.

### Plant Disease Suppression

The inoculation of plants with the selected bacterial isolates significantly improved plant growth and plant photosynthetic parameters compared with the non-inoculated control in plants infected with *P. capsici* (**Figure 8**). Beneficial bacteria secrete various types of volatile and involatile signal compounds that play an important role in plant growth regulation, development, and systemic resistance (Kai et al., 2016) and they can be antagonistic to plant pathogens (Sharifi and Ryu, 2016). Similar studies have shown that PGPR strains applied as seed treatment were found to significantly reduce disease severity of *Phytophthora* crown rot in cucumber plants (Islam et al., 2016). The qPCR results further confirmed the initial phenotypic observations and the plant physiological data in the previous sections. Upon the application of beneficial bacteria, the pathogen load in the root of chili was decreased. This result is in agreement to a recent study in which rhizobacteria isolates exhibited plant-growth promotion and biological control of multiple plant diseases through the mechanism of antagonism and systemic resistance (Liu et al., 2018). Silvar et al. (2005) stated that the level of resistance exhibited by plants against pathogens depends on the degree of coordination between different defense responses. They investigated the host–pathogen interactions of four cultivars of pepper with altered susceptibility to *P. capsici*. The amount of pathogen DNA quantified by qPCR in each pepper genotype was correlated to the level of pathogen virulence and host susceptibility to *Phytophthora* root rot. A similar study by Barriuso et al. (2008) provided evidence that four PGPR isolates enhanced protection against foliar pathogen *Pseudomonas syringae* DC3000 in *A. thaliana* Col-0 and significantly increased growth and photosynthesis both in normal and stress conditions. Co-inoculation with *Rhizobium* and *Pseudomonas* improved the photosynthetic rate, CO_2_ assimilation rate and chlorophyll content of mung beans (Ahmad et al., 2013). The genotype of different plant varieties is critical for specific plant–microbe interactions. For *Arabidopsis*, up to fourfold more biomass in the presence of *Pseudomonas simiae* WCS417r was measured for some ecotypes, while no improvements were found in others (Khalid et al., 2004). In the present study, by observing the root architecture, plants inoculated with bacterial isolates had more lateral roots compared to uninoculated, uninfected or *P. capsici*-infected chili plants, harboring more rhizosphere soil (**Supplementary Figure S5**). Further investigations will be required to determine if the inocula trigger ISR, act directly on the oomycetes or if both events occur in the observed results.

### Isolation of Putative Anti-oomycete Bacterial Compounds

Soil bacteria produce a large number of secondary metabolites with many different physiochemical and biological properties (Tyc et al., 2017). The biocontrol activity of the isolates may be attributed to the ability to produce a vast array of structurally different secondary metabolites, which can suppress harmful microbes living within the plant rhizosphere. There is a possibility that the inhibition observed in dual cultures is a result of the production of secondary anti-oomycete compounds. Most of the compounds identified in this study are common biologically active DKPs, in the bacterial extracts (**Figure 10**), with some of them exhibited antimicrobial activities against different pathogens.

Diketopiperazines appear to confer a strong inhibition upon different stages of *P. capsici* and *Phytophthora colocasiae* life cycle; no new mycelium was observed and formation of sporangium was completely inhibited in a previous study (Kumar and Nambisan, 2014). In another study, the DKP (2,5-diketopiperazine) cyclo (l-Pro-l-Tyr) was shown to be effective in killing the sporangia of plant pathogenic oomycetes, *Phytophthora infestans* and *Plasmopara viticola*, *in vitro* and on tomato leaves (Puopolo et al., 2014).

The bacterium *Pseudoalteromonas* sp. associated with the *octocoral* L. *alba* has been found to produce cyclo-(L-Leu-L-Pro) which showed antagonistic activity against *Vibrio* sp. and *Bacillus subtilis* (Martínez-Luis et al., 2011). The antifungal activity of cyclo (L-Pro-L-Leu), cyclo (D-Pro-L-Leu), and cyclo (L-Pro-D-Leu) from TSB medium was also reported against pathogenic fungi and bacteria (Nishanth Kumar et al., 2012). This compound was also suggested as a promising alternative to chemical preservatives as a potential biopreservative which prevents fungal growth and mycotoxin formation in food and feed (Kumar et al., 2013). Cyclo (L-Leu-L-Pro) 4, cyclo (L-Phe-L-Pro) isolated from *Aspergillus fumigatus* were found to inhibit the growth of *Staphylococcus aureus* and *Micrococcus luteus* using *in vitro* assays (Furtado et al., 2005). Cyclo (Leu-Leu) produced by *Lactobacillus plantarum* AF1 isolated from kimchi was found to have antifungal activity against *Aspergillus flavus* and may be a promising alternative to chemical preservatives as a potential biopreservative which prevents fungal spoilage and mycotoxin formation in food and feed (Yang and Chang, 2010). Cyclo (Pro-Phe) was also found to have a significant effect on the antifungal activity of *B. amyloliquefaciens* Q-426 (Wang et al., 2017), and previous studies showed a positive response to biosensors which were used to detect signal molecules (Chhabra et al., 1999; Degrassi et al., 2002). 5,10-diethoxy-2,3,7,8-tetrahydro-1H,6H-dipyrrolo[1,2-a:1′,2′-d]pyrazine was found in essential oil of *Trigonella foenum-graecum* that showed antibacterial activity against Gram positive and Gram negative bacteria (Nahar et al., 2016). 3-Butyl-6-methylpiperazine-2,5-dione was identified as antimicrobial compounds extracted from *B. subtilis* (PSB5) against *Fusarium oxysporum* f. sp. *gerberae* (Suneeta et al., 2016).

Further studies are needed to verify whether each of these compounds exhibits anti-oomycete activities in the field and if their biosynthesis is regulated in response to *Phytophthora* infection. Furthermore, GC-MS analysis can only test heat-stable compounds. Thus, there is a possibility that other compounds with similar activities have been degraded at high temperatures. The identification of the actual compound/s causing the oomycete inhibition would be crucial in the preparation of a biocontrol formulation, which could be then used in the field.

### Potential Application of the Isolates and Compounds as Biocontrol Agents

The beneficial bacteria identified in this study can potentially be used in glasshouse and field trials as agricultural inoculants for testing improved plant productivity. *Bacillus* strains have been shown to be effective as biofertilizers and biocontrol agents in agriculture (Yao et al., 2006; Borriss, 2011; Wang et al., 2013). The use of these bacteria has proven to be effective to suppress pathogenic microbes, encourage beneficial effects on plant growth and facilitate nutrient accessibility and assimilation (Sharma et al., 2009; Compant et al., 2013; Bach et al., 2016; van Lenteren et al., 2018). Several factors determine the practicality of biocontrol agents in the field (Singh et al., 2016). For example, issues related to long-term storage and competition from indigenous soil microbiota have hindered the development of previous commercial inoculants. This study utilized sterilized potting mix as soil, where the microbial load was likely substantially lower than in field soil. Future work investigating the applicability of the isolates detailed in this study under field conditions are required. For example, isolate UQ202 could be tested in chili pepper fields by applying these bacteria to soil in irrigation lines.

## Conclusion

Future studies should focus on identifying and characterizing compounds synthesized by the biocontrol and growth-promoting agents and their application in plants for better yields and plant protection. Finding and incorporating new mechanisms of host resistance is the key to effective management of plant diseases. Hence, in the quest to enhance plant yield and soil fertility as well as to reduce the harmful impacts of chemical fertilizers in the environment, there is growing interest to exploit beneficial bacteria for better productivity and sustainability of agricultural systems. This can be accomplished by understanding the mechanisms involved during the interaction of antagonists and the pathogens.

## Author Contributions

SFS-A-R conducted all experiments and contributed to the design and implementation of the research, to the analysis of the results and the writing of this manuscript. LC and PS contributed to the writing and proofreading of the manuscript. EC performed the GC-MS analysis to elucidate compounds present in the selected isolates that harbor potential biocontrol activity. YX assisted with performing the experiments to assay plant growth promoting attributes of the putative PGPR. TW assisted with designing the visual presentation of the hierarchically clustered heat-map.

## Conflict of Interest Statement

The authors declare that the research was conducted in the absence of any commercial or financial relationships that could be construed as a potential conflict of interest.

## Footnotes

1 https://blast.ncbi.nlm.nih.gov/Blast.cgi

## Supplementary Material

FIGURE S1 Standard curve generated by plotting threshold cycle (Ct) numbers against a 10-time dilution series of *P. capsici* genomic DNA, starting with 400 ng. The efficiency of the curve was 100%, with a correlation coefficient 0 to 9.963 and a slope −3.316.

FIGURE S2 Ethidium bromide-stained 1% agarose gel showing amplicons of the full-length 16S rDNA gene.

FIGURE S3 Nitrogen-fixation ability of the isolates was determined by the change of color of NFB medium from green to blue after 4-day incubation. Red circles indicate the radial bacterial growth pattern.

FIGURE S4 Chromeazurol S agar test, for siderophore detection in a plate culture of isolate UQ154, UQ156, and UQ202. The culture medium with CAS blue dye contains halos indicating the presence of siderophores.

FIGURE S5 We noticed higher root biomass in plants inoculated with bacterial isolates *B. amyloliquefaciens* (UQ154), *B. velezensis* (UQ156), and *Acinetobacter* (UQ202) compared to uninoculated (control) or *P. capsici*-infected chilli plants, harboring more rhizosphere soil.

TABLE S1 16S rDNA gene matches of the bacterial isolates characterized in this study.
