# Supplementary figures and images for: Identification of Soil Bacterial Isolates Suppressing Different Phytophthora spp. and Promoting Plant Growth
Source: Front Plant Sci. 2018 Oct 18;9:1502. doi: 10.3389/fpls.2018.01502 (PMC6201231; doi:10.3389/fpls.2018.01502)

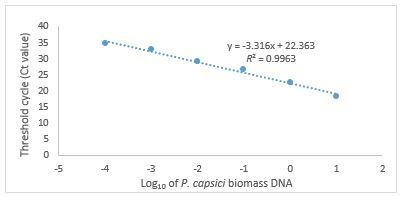

Supplement: FIGURE S1 — Standard curve generated by plotting threshold cycle (Ct) numbers against a 10-time dilution series of P. capsici genomic DNA, starting with 400 ng. The efficiency of the curve was 100%, with a correlation coefficient 0 to 9.963 and a slope −3.316. [file Image_1.JPEG]

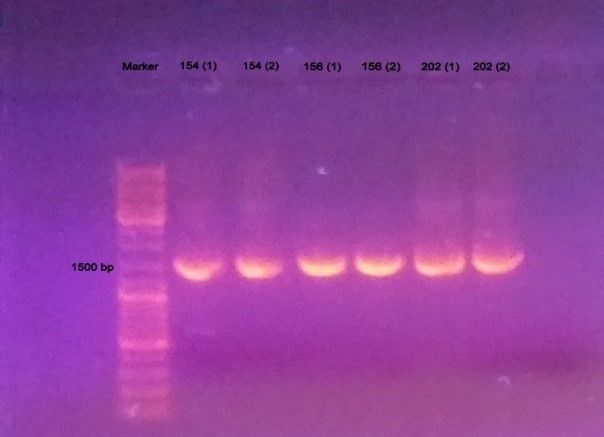

Supplement: FIGURE S2 — Ethidium bromide-stained 1% agarose gel showing amplicons of the full-length 16S rDNA gene. [file Image_2.JPEG]

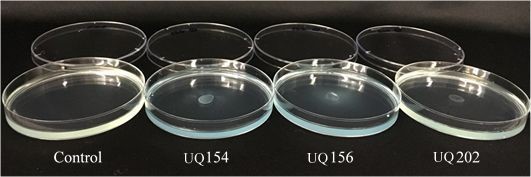

Supplement: FIGURE S3 — Nitrogen-fixation ability of the isolates was determined by the change of color of NFB medium from green to blue after 4-day incubation. Red circles indicate the radial bacterial growth pattern. [file Image_3.JPEG]

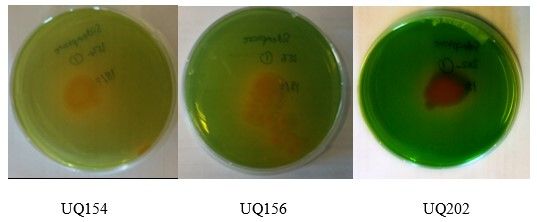

Supplement: FIGURE S4 — Chromeazurol S agar test, for siderophore detection in a plate culture of isolate UQ154, UQ156, and UQ202. The culture medium with CAS blue dye contains halos indicating the presence of siderophores. [file Image_4.JPEG]

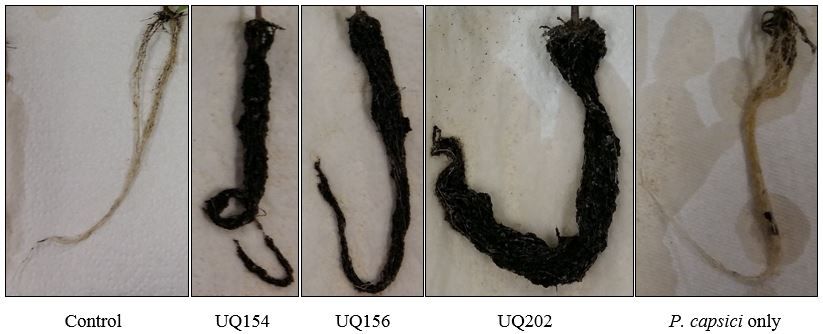

Supplement: FIGURE S5 — We noticed higher root biomass in plants inoculated with bacterial isolates B. amyloliquefaciens (UQ154), B. velezensis (UQ156), and Acinetobacter (UQ202) compared to uninoculated (control) or P. capsici-infected chilli plants, harboring more rhizosphere soil. [file Image_5.JPEG]
