# Supplementary material for: Identification of Soil Bacterial Isolates Suppressing Different Phytophthora spp. and Promoting Plant Growth
Source: Front Plant Sci. 2018 Oct 18;9:1502. doi: 10.3389/fpls.2018.01502 (PMC6201231; doi:10.3389/fpls.2018.01502)
Supplement: TABLE S1 — 16S rDNA gene matches of the bacterial isolates characterized in this study. [file Table_1.DOCX]

**Table S1:** 16S rDNA gene matches of the bacterial isolates characterized in this study

| Isolate ID | Accession | Highest Scoring BLAST Hit | Best Hit Accession | Sequence Identity (%) |
| --- | --- | --- | --- | --- |
| 141 | - | *Pseudomonas koreensis* strain IHB B 7199 | KJ767342.1 | 93 |
| 145 | - | *Pseudomonas putida* strain DNCA01 | KF030905.1 | 94 |
| UQ154 | MG877642 | *Bacillus amyloliquefaciens* | JQ765431.1 | 99 |
| 155 | - | *Alcaligenaceae* bacterium strain DZQ19 | HQ143587.1 | 95 |
| UQ156 | MG877641 | *Bacillus velezensis* | CP019040.1 | 99 |
| UQ202 | MG877638 | *Acinetobacter* sp. | HF563569.1 | 99 |
| 212 | MH725611 | *Pseudomonas veronii* strain E02 | KT326185.1 | 98 |
| 214 | MH725599 | *Pseudomonas* sp. strain SB1246 | MG491539.1 | 99 |
| 222 | MH725612 | *Pseudomonas koreensis* strain OFM42 | MH542340.1 | 99 |
| 224 | MH725613 | *Pseudomonas granadensis* strain 26C3 | MG269614.1 | 99 |
| 231 | MH725614 | *Pseudomonas* sp. strain SB1246 | MG491539.1 | 99 |
| 233 | MH725600 | *Pseudomonas* sp. strain SB851 | MG491626.1 | 99 |
| 240 | MH725615 | *Pseudomonas* sp. 3-28(2010) | HM489948.1 | 99 |
| 242 | - | *Pseudomonas migulae* strain AN-1 | KF857261.1 | 99 |
| 243 | - | *Pseudomonas migulae* strain AN-1 | KF857261.1 | 99 |
| 245 | - | *Pseudomonas migulae* strain AN-1 | KF857261.1 | 99 |
| 252 | - | *Pseudomonas* sp. RC202 | KJ534435.1 | 99 |
| 253 | MH725616 | *Pseudomonas* sp. strain 62Zx | KX108971.1 | 99 |
| 255 | - | *Pseudomonas putida* strain DNCA01 | KF030897.1 | 99 |
| 256 | - | *Pseudomonas* sp. CK8-10 | JN195810.1 | 99 |
| 260 | MH725617 | *Pseudomonas koreensis* strain OFM42 | MH542340.1 | 97 |
| 262 | MH725601 | *Pseudomonas* sp. A10.1(2011) | HQ412501.1 | 98 |
| 272 | MH725618 | *Pseudomonas grimontii* strain 4G483 | KY939749.1 | 99 |
| 278 | MH725618 | *Pseudomonas rhodesiae* strain 67B5 | MG269719.1 | 99 |
| 280 | MH725620 | *Pseudomonas grimontii* strain 4G483 | KY939749.1 | 99 |
| 282 | MH725621 | *Pseudomonas rhodesiae* strain 67B5 | MG269719.1 | 99 |
| 287 | MH725622 | *Pseudomonas rhodesiae* strain 67B5 | MG269719.1 | 99 |
| W26 | - | W26* | - | - |
| W27 | MH725602 | *Enterobacter* sp. MLB03 | JQ765414.1 | 99 |
| W28 | MH725603 | *Kosakonia cowanii* strain Bb4 | LN907847.2 | 97 |
| W29 | - | W29* | - | - |
| W32 | MH725604 | *Enterobacter* sp. MLB03 | JQ765414.1 | 98 |
| W34 | MH725605 | *Enterobacter* sp. MLB27 | JQ765423.1 | 99 |
| W35 | MH725606 | *Enterobacter* sp. HT-Z60 | KJ526920.1 | 99 |
| W36 | - | W36* | - | - |
| W37 | MH725623 | *Salmonella* sp. strain PYRL10 | MF458869.1 | 98 |
| W38 | MH725607 | *Enterobacter* sp. MLB03 | JQ765414.1 | 99 |
| W40 | MH725608 | *Pseudomonas* sp. P-W-1 | KC991325.1 | 99 |
| W41 | - | W41* | - | - |
| W42 | MH725624 | *Enterobacter* sp. HT-Z60 | KJ526920.1 | 99 |
| W43 | MH725609 | *Kosakonia cowanii* strain Esp_Z | CP022690.1 | 99 |
| W44 | - | W44* | - | - |
| W45 | - | W45* | - | - |
| W46 | MH725625 | *Enterobacter* sp. HT-Z60 | KJ526920.1 | 99 |
| W47 | MH725610 | *Enterobacter* sp. HT-Z60 | KJ526920.1 | 100 |
| W48 | MH725626 | *Enterobacter* sp. HT-Z60 | KJ526920.1 | 99 |
| W49 | MH725627 | *Kosakonia* sp. strain B1-5 | MH051262.1 | 99 |
| W50 | - | W50* | - | - |

* indicates that strain could not be identified due to either sample mortality or issues with sequencing.
